# Magnetic nanoparticles hyperthermia in a non-adiabatic and radiating process

**DOI:** 10.1038/s41598-021-91334-9

**Published:** 2021-06-04

**Authors:** C. A. M. Iglesias, J. C. R. de Araújo, J. Xavier, R. L. Anders, J. M. de Araújo, R. B. da Silva, J. M. Soares, E. L. Brito, L. Streck, J. L. C. Fonseca, C. C. Plá Cid, M. Gamino, E. F. Silva, C. Chesman, M. A. Correa, S. N. de Medeiros, F. Bohn

**Affiliations:** 1 Departamento de Física, Universidade Federal do Rio Grande do Norte, 59078-900 Natal, RN Brazil; 2 Departamento de Física, Universidade do Estado do Rio Grande do Norte, 59610-090 Mossoró, RN Brazil; 3 POLYMAT, Departamento de Química Aplicada, Facultad de Ciencias Químicas, University of the Basque Country UPV/EHU, Joxe Mari Korta Zentroa, Tolosa Hiribidea 72, 20018 Donostia-San Sebastián, Spain; 4 Instituto de Química, Universidade Federal do Rio Grande do Norte, 59078-970 Natal, RN Brazil; 5 Curso de Farmácia, Faculdade Maurício de Nassau, 59080-400 Natal, RN Brazil; 6 Departamento de Física, Universidade Federal de Santa Catarina, 88040-900 Florianópolis, SC Brazil

**Keywords:** Magnetic properties and materials, Nanoparticles, Characterization and analytical techniques

## Abstract

We investigate the magnetic nanoparticles hyperthermia in a non-adiabatic and radiating process through the calorimetric method. Specifically, we propose a theoretical approach to magnetic hyperthermia from a thermodynamic point of view. To test the robustness of the approach, we perform hyperthermia experiments and analyse the thermal behavior of magnetite and magnesium ferrite magnetic nanoparticles dispersed in water submitted to an alternating magnetic field. From our findings, besides estimating the specific loss power value from a non-adiabatic and radiating process, thus enhancing the accuracy in the determination of this quantity, we provide physical meaning to a parameter found in literature that still remained not fully understood, the effective thermal conductance, and bring to light how it can be obtained from experiment. In addition, we show our approach brings a correction to the estimated experimental results for specific loss power and effective thermal conductance, thus demonstrating the importance of the heat loss rate due to the thermal radiation in magnetic hyperthermia.

## Introduction

Magnetic hyperthermia (MHT) corresponds to the effect that exploits the heat generated by magnetic nanoparticles (MNPs) when submitted to an alternating magnetic field (AMF). In recent decades, magnetic nanoparticles (or ferrofluid) hyperthermia has received increasing interest due to the possibility of its application as a thermal therapy in clinical trials for the treatment of cancers and other diseases, as well as in the process of thermally activated drug delivery under AMF^1–10^. Within this context, specific features of the magnetic nanoparticles dispersed in a carrier liquid are essential, being explored to perform the drug delivery and/or destroy ill cells by heating.

The magnetic hyperthermia effect has been extensively investigated both theoretically and experimentally. In magnetic nanoparticles (or ferrofluid) hyperthermia, the potential of a given magnetic material is in general evaluated through the specific loss power (*SLP*), often also denoted by the so-called specific absorption rate (*SAR*)^11^. *SLP* is simply the power generated per unit mass of the magnetic material^12^. This quantity, described in terms of a linear response theory^13^, is notably a function of both, material/sample properties (for instance, saturation magnetization, magnetic susceptibility, magnetic anisotropy, magnetization relaxation times, particle size, shape of the nanoparticle, particle concentration, volume and liquid viscosity) and experiment conditions (such as waveform, frequency and amplitude of the alternating magnetic field). For this reason, hyperthermia also appears as an important tool to provide insights on the magnetic behavior of magnetic nanoparticles, contributing specifically to the understanding of the fundamental physics associated to the magnetization dynamics in such systems with reduced dimensions.

In a typical hyperthermia experiment, the evolution of the temperature of the nanoparticles or the ferrofluid with time is probed. From this measurement, *SLP* is commonly quantified by standard calorimetric methods in which a quasi-adiabatic regime is assumed, i.e. the nanoparticles or the ferrofluid behave as a quasi-adiabatic system whose energy is absorbed by the magnetic material at a constant rate^2,4,12,14–18^. Within this picture, just the slope of the temperature curve during a short time interval after applying AMF is analysed. Hence, this procedure brings intrinsic uncertainties, as well as it frequently underestimates the *SLP* value for a suspension of MNPs^17,19–21^. Going beyond, non-adiabatic calorimetric methods not taking into account radiation effects have also been employed for the *SPL* estimate^19–23^, thus providing an enhancing the accuracy in the determination of this quantity. From this perspective, the whole temperature curve, considering a longer time interval after applying AMF, is explored. However, while the quasi-adiabatic model and the non-adiabatic and non-radiating approach have been widely investigated, the same effort was not intended to the analysis of *non-adiabatic and radiating* calorimetric methods to this end. As a consequence, many questions on the magnetic heating power of MNPs and the determination of *SLP* are still open. Among them, perhaps the most remarkable doubt on the issue resides in the influence that the energy losses to the environment may have on the magnetic nanoparticles (or ferrofluid) hyperthermia response. In particular, the answer for this issue directly impacts the technological fields of engineering and biomedicine, given that, in applications, a suspension of MNPs is commonly not insulated. In this sense, we understand that a theoretical approach that considers parameters related to the interaction of the magnetic fluid with the environment, including thermal radiation, becomes needed, thus providing further accurate estimates of *SLP* for suspensions of magnetic nanoparticles under AMF in non-adiabatic conditions.

In this article, we investigate the magnetic hyperthermia in suspensions of MNPs. Specifically, we propose a theoretical approach to magnetic hyperthermia from a thermodynamic point of view. The model allows us to obtain the *SLP* value from a non-adiabatic and radiating process, thus enhancing the accuracy in the determination of this quantity. Further, we provide physical meaning to a parameter found in literature that still remained not fully understood, the effective thermal conductance, and bring to light how it can be obtained experimentally. To test the robustness of the approach, we perform hyperthermia experiments and analyse the thermal behavior of magnetite and magnesium ferrite MNPs dispersed in water submitted to an AMF. Then, we demonstrate the thermal radiation losses cannot be neglected in such process.

## Results

### Theoretical approach

Here, to investigate the specific loss power of magnetic nanoparticles hyperthermia, we focus on the temperature response of MNPs dispersed in a fluid submitted to an AMF. To this end, we employ a theoretical approach based basically on thermodynamics concepts and, therefore, without the need of a microscopic description of the system.

### Mimicking an adiabatic system

We start presenting the well-known adiabatic model for magnetic hyperthermia^2,4,12,14–18^, with its assumptions and limitations.

In MHT, the heating effect of a magnetic fluid is a result of absorbing energy from the AMF and converting it into a raise of the internal energy and/or heat by eddy current losses^16^, as well as quasi-static^24^ and dynamic^16,25,26^ hysteresis losses. Generally, magnetic fluids exhibit low electrical conductivity, in a sense the eddy current losses do not arise and can be in principle neglected. Next, quasi-static hysteresis losses are attributed to ferromagnetic/ferrimagnetic features of the particles, and they are directly related to the magnetization reversion during the magnetization process in such magnetic materials. At last, dynamic hysteresis losses are yet ascribed to ferromagnetic/ferrimagnetic materials, as well as are also extended to superparamagnetic compounds^22^. For this latter kind of loss, it is worth remarking that there are two distinct mechanisms by which the magnetization of magnetic fluids relaxes after the magnetic field is removed. The first one is associated to the so-called Brownian relaxation^25,26^. In this case, the particle moves freely within the suspension, and the relaxation takes place due to the reorientation of the whole particle, being a result of the viscous friction between the rotating particle and surrounding medium^16^. The second mechanism in turn is connected to the Néel relaxation^25,26^. Specifically, it consists in the reversion of the magnetic moment within the particle, once the magnetic moment overcomes an energy barrier due to the uniaxial anisotropy.

Despite the diversity in essence, the losses in all cases come from the irreversible work undergone by the suspension due to interaction effects of the magnetic particles with the AMF. In order to quantify the variation of the internal energy of the suspension due to the irreversible work associated with the interaction of the magnetic field with the system, we take into account the general Principle of Energy Conservation — In an energetically isolated system, the total energy remains constant during any change which may occur in it. When adapted for thermodynamic processes, it is expressed by the First Law of Thermodynamics, given by

1 $$\begin{aligned} \Delta U_{susp} = W - Q_{susp}, \end{aligned}$$

where in our context $\Delta U_{susp}$ is the variation of the internal energy of the suspension, *W* is the irreversible work *undergone by the suspension*, and $Q_{susp}$ is the heat *lost by the suspension*. Here, we can split the work *W* into two components; the first, depicted by $W_{mag}$, corresponds to the work *undergone by the suspension due to the interaction of the magnetic nanoparticles with the alternating magnetic field*; the second one, $W_{mec}$ is the mechanical work *done on the suspension*.

For an adiabatic ($Q_{susp} = 0$) and isochoric ($W_{mec} = 0$) process, $\Delta U_{susp}$ can be written simply as

2 $$\begin{aligned} \Delta U_{susp} = W_{mag} = C_{susp} \Delta T , \end{aligned}$$

where $\Delta T$ is the temperature variation of the system, i.e. the suspension, and $C_{susp}$ is the heat capacity of the suspension, which can be expressed in a generalized form as

3 $$\begin{aligned} C_{susp} = \sum _{j}^N m_{j} c_{j} , \end{aligned}$$

in which $m_{j}$ and $c_{j}$ are the mass and specific heat of the $j-th$ constituent (magnetic nanoparticles and fluid) of the suspension, respectively, and *N* is the total number of constituents in the suspension.

The specific loss power, as aforementioned, is defined as the power generated ($W/\Delta t$), where $\Delta t$ is a time interval, per unit mass of the magnetic material ($m_{np}$). Hence, considering Eq. (2) mimicking a system in an adiabatic and isochoric process, *SLP* may be expressed as

4 $$\begin{aligned} SLP = \dfrac{1}{m_{np}} \dfrac{W_{mag}}{\Delta t} =\frac{\left( \Delta U_{susp}/\Delta t\right) }{ m_{np}}= \dfrac{1}{m_{np}} C_{susp} \dfrac{\Delta T}{\Delta t} . \end{aligned}$$

Remarkably, Eq. (4) has been addressed and employed in numerous works found in literature^2,4,12,14–18^. However, it is worth pointing out that this first approach to estimate *SLP* has validity only in the quasi-adiabatic regime, i.e. when the system is insulated and its temperature is considered varying as a linear function with time. This assumption is a key factor that may affect the results, in a sense we should look with care at the *SLP* findings. In addition, the fact that the suspension of MNPs is not insulated in applications makes this assumption a limitation of the adiabatic approach.

### Approaching a system in non-adiabatic conditions

Keeping in mind that the suspension of magnetic nanoparticles often interacts with the environment in applications and even in experiments, this fact cannot be neglected in a model addressing magnetic hyperthermia. Here, we propose a theoretical approach based on thermodynamics concepts that takes into account this interaction, thus improving the *SLP* estimates. Specifically, we assume the interaction between system and environment is embedded in the contribution of the heat loss in the First Law of Thermodynamics, i.e. the $Q_{susp}$ term in Eq. (1). Hence, we handle with a suspension of magnetic nanoparticles submitted to an alternating magnetic field in *a non-adiabatic and radiating process*.

**Figure 1:**
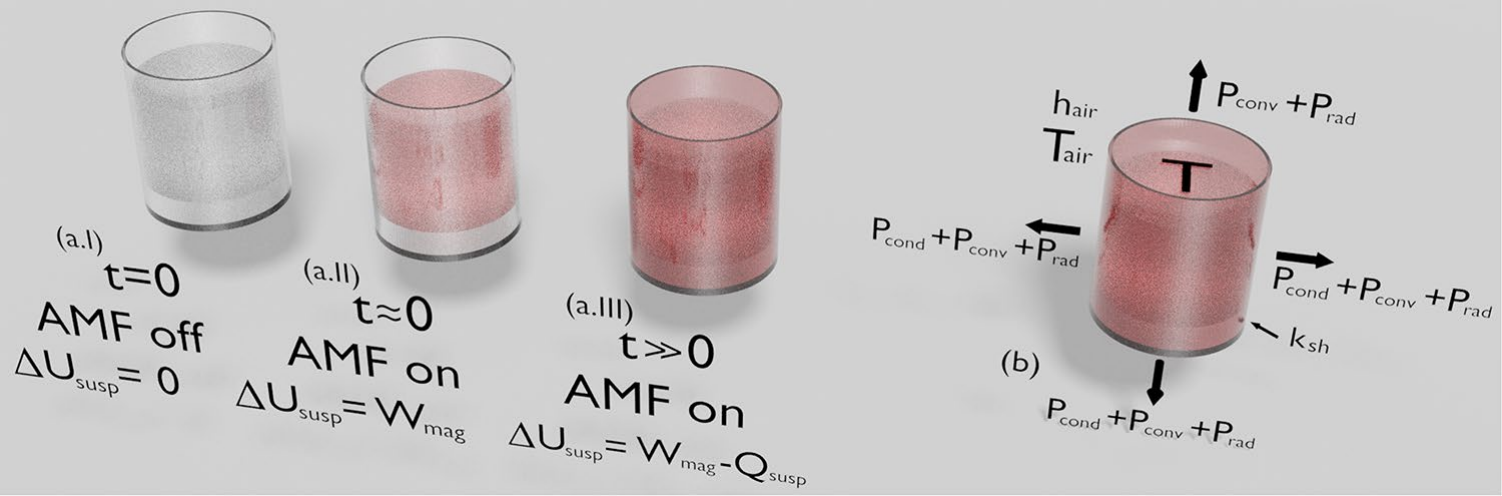
Schematic representation of our theoretical system—a suspension of magnetic nanoparticles inside a cylindrical sample holder, which is submitted to an alternating magnetic field. Suspension (**a.I**) in thermal equilibrium with the environment, (**a.II**) in an adiabatic regime during a short time interval just after the AMF is turned on, and (**a.III**) in the non-adiabatic regime. (**b**) Definitions of some quantities employed in our theoretical approach. Here, we consider *T* as the temperature of the suspension, and $P_{cond}$ as the heat loss rate due to the process of conduction through the walls of the sample holder, with $\kappa _{sh}$ denoting the thermal conductivity of the sample holder; $P_{conv}$ corresponds to the heat loss rate associated to the convection process of heat transfer from the outer surface of the sample holder and the upper surface of the sample, both surrounded by air; $h_{air}$ is the heat transfer coefficient of the air, and $T_{air}$ is the temperature of the environment; and finally $P_{rad}$ is heat loss rate due to the thermal radiation.

To this end, we start our approach to the magnetic hyperthermia from a system in non-adiabatic and non-radiating conditions. Generally, our system consists of magnetic nanoparticles dispersed in a fluid submitted to an alternating magnetic field. The suspension of magnetic nanoparticles is inside a sample holder, which plays as boundaries that split it from the environment, as we can see in Fig. 1.

In a MHT experiment, first, while the AMF is off, the system is in thermal equilibrium with the environment (Fig. 1a.I). As soon as the magnetic field is turned on, it acts on the system, and a work $W_{mag}$ is done on the suspension. In particular, at this stage, an adiabatic process is assumed; and this total irreversible work undergone by the suspension is converted to internal energy of the system, what is evidenced through an increase of the system temperature (Fig. 1a.II). We understand that the heat loss $Q_{susp}$ may be neglected during a quite-short time interval; and, therefore, the approach for the system in the quasi-adiabatic regime becomes enough. Hence, Eq. (4) may be used carefully. However, after this interval in which the temperature varies linearly with time, the quasi-adiabatic approximation loses its validity. In this case, a fraction of the energy drawn from the magnetic field is converted into heat loss as well, giving rise to the energy exchange between suspension and environment (Fig. 1a.III).

Given all the stated above, we also start our approach from the the First Law of Thermodynamics, Eq. (1). Here, although we assume a non-adiabatic regime, the process yet remains to be isochoric, without thermal expansions and/or mechanical work done on the suspension. Then,

5 $$\begin{aligned} \Delta U_{susp} = W_{mag} - Q_{susp}, \end{aligned}$$

where, just to remember, $\Delta U_{susp}$ is the variation of the internal energy of the suspension, $W_{mag}$ is the irreversible work *undergone by the suspension due to the interaction of the magnetic particles with the alternating magnetic field*, and $Q_{susp}$ is the heat *lost by the suspension*.

From the differentiation of Eq. (5) with respect to time, we may express *SLP* as

6 $$\begin{aligned} SLP = \dfrac{1}{m_{np}} C_{susp} \frac{dT}{dt} + \frac{1}{m_{np}} \frac{dQ_{susp}}{dt}. \end{aligned}$$

Notice that the most suitable definition for the specific loss power in the generalized case, i.e. Eq. (6), is actually the total irreversible work rate per magnetic material mass undergone by the suspension. As a consequence, Eqs. (4) and (6) are similar, except by the second term in the definition for the *SLP* in the non-adiabatic regime. This latter denotes the dependence of *SLP* with the rate of heat loss of the system to the environment, which we define here as

7 $$\begin{aligned} P \equiv \frac{dQ_{susp}}{dt}. \end{aligned}$$

We first address here the heat loss rate due to the process of conduction through the walls of the sample holder. Then, taking into account the Fourier’s Law^27^, it may be written as

8 $$\begin{aligned} P_{cond} = -\kappa _{sh} A_{sh,int} \dfrac{dT}{dr}, \end{aligned}$$

where $\kappa _{sh}$ and $A_{sh,int}$ are the thermal conductivity and the inner surface area of the sample holder, respectively. It is worth remarking that the heat loss rate is assumed to be normal to the surfaces of the system (See Fig. 1b). Additionally, for sake of simplicity, we use a convenient form of sample holder, i.e. cylindrical form. As a consequence, the variable *r* denoting the radial distance, as well as *z* expressing the height in cylindrical coordinates, changes in a direction normal to the system surfaces and to the heat reservoir through the walls of the sample holder. This fact simplifies the solve of Eq. (8), without loss of generality.

Then, from Eq. (8), under the boundary conditions of $T(R_{int}) = T(z_{bottom,int}) = T$, $T(R_{ext}) = T_{ext}$ and $T(z_{bottom,ext}) = T_{ext}$, the heat loss rate due to the process of conduction through the walls of the sample holder may be expressed by

9 $$\begin{aligned} P_{cond} = \kappa _{sh} \left( \dfrac{A_{side}}{R_{ext} \ln (R_{ext}/R_{int})} + \dfrac{A_{bottom}}{L}\right) (T - T_{ext}), \end{aligned}$$

where $A_{side}$ and $A_{bottom}$ are the lateral and bottom inner areas of the sample holder, respectively, $L= z_{bottom,ext} - z_{bottom,int}$ is the thickness of the wall, $R_{int}$ the inner radius and $R_{ext}$ the outer radius of the sample holder, $T_{ext}$ is the temperature of the outer surface of the sample holder, and *T* is the temperature of the suspension.

Next, we address the heat loss rate due to the convection process of heat transfer from the outer surface of the sample holder and the upper surface of the sample, both surrounded by air. In this case, by means of the Newton’s Law of cooling^28^, it can be expressed as

10 $$\begin{aligned} P_{conv} = h_{air} A_{sh,ext} (T_{ext} - T_{air}) + h_{air} A_{top} (T - T_{air}), \end{aligned}$$

where $A_{top}$ is the upper surface area of the sample, $A_{sh,ext}$ is the outer surface area of the sample holder, $h_{air}$ is the heat transfer coefficient of the air, and $T_{air}$ is the temperature of the environment. It is worth mentioning that the environment, i.e. the air, is assumed to have properties of heat reservoir, exhibiting $\frac{d T_{air}}{d t} = 0$.

From Eqs. (9) and (10), we can define

11 $$\begin{aligned}&\epsilon _{sh} \equiv \kappa _{sh} \left( \dfrac{A_{side}}{R_{ext} \ln (R_{ext}/R_{int})} + \dfrac{A_{bottom}}{L}\right) , \end{aligned}$$

12 $$\begin{aligned}&\epsilon _{air,surf} \equiv h_{air} A_{sh,ext}, \end{aligned}$$

and

13 $$\begin{aligned} \epsilon _{air,top} \equiv h_{air} A_{top}. \end{aligned}$$

Here, $\epsilon _{sh}$ represents the thermal conductance of the sample holder, $\epsilon _{air,surf}$ is the thermal conductance associated to the convection of the air on the outer surface of the sample holder, and $\epsilon _{air,top}$ corresponds to thermal conductance associated to the convection of the air on the interface sample/air.

**Figure 2:**
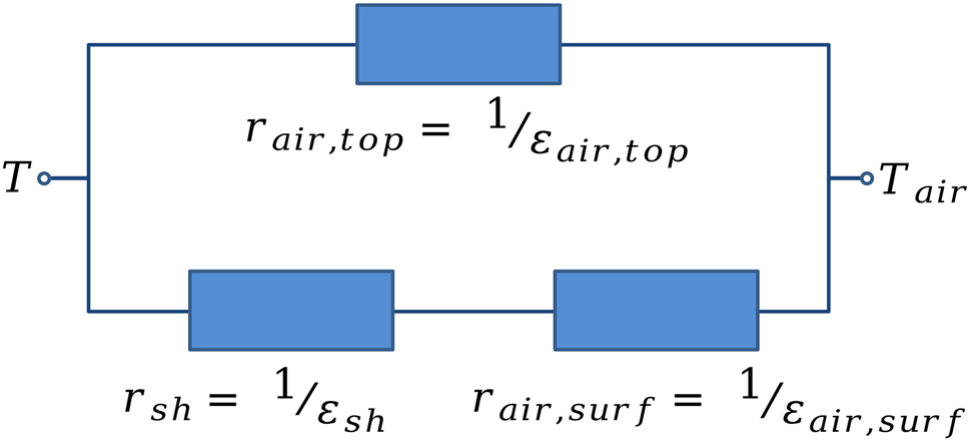
Schematic representation of the equivalent circuit employed to the obtainment of the effective thermal conductance, taking into account the conduction and convection processes contributing to the energy exchange between suspension and environment. Here, we represent $r_{air,top} =1/\epsilon _{air,top}$ as the thermal resistance of the air at the interface sample/air, $r_{air,surf} = 1/\epsilon _{air,surf}$ as thermal resistance associated to the convection of the air on the outer surface of the sample holder, and $r_{sh} = 1/\epsilon _{sh}$ as thermal resistance of the sample holder.

In analogy with electric circuits, taking into account just the conduction and convection processes contributing to the energy exchange between suspension and environment, the effective thermal conductance can be obtained by mean of the parallel association of the thermal resistance of the air at the interface sample/air, $r_{air,top} =1/\epsilon _{air,top}$, with the series association of the thermal resistance of the sample holder, $r_{sh} = 1/\epsilon _{sh}$, and the thermal resistance associated to the convection of the air on the outer surface of the sample holder, $r_{air,surf} = 1/\epsilon _{air,surf}$. Figure 2 depicts a sketch of the equivalent circuit employed to the obtainment of the effective thermal conductance.

As a result, the rate of heat loss of the system to the environment through the conduction and convection processes, named here as $P_{c},$ may be written as

14 $$\begin{aligned} P_{c} = \epsilon (T - T_{air}), \end{aligned}$$

where

15 $$\begin{aligned} \epsilon = \left( \frac{\epsilon _{sh} \epsilon _{air,surf}}{\epsilon _{sh} + \epsilon _{air,surf}}\right) + \epsilon _{air,top} \end{aligned}$$

is the parameter we define here as the effective thermal conductance into the surrounding of the sample. Notice that *T* is the quantity probed in MHT experiments.

At temperatures between 300 and 320 K, within the range required for biological applications, as well as for temperature right above this limit, the heat loss rate due to the radiation is often neglected^20,23^. Thereby, from Eqs. (6) and (14), we obtain

16 $$\begin{aligned} SLP = \dfrac{1}{m_{np}} C_{susp} \dfrac{dT}{dt} + \dfrac{1}{m_{np}} \epsilon (T-T_{air}). \end{aligned}$$

The solution for the differential equation in the heating process, under the condition $T(0) = T_{air}$ depicting the suspension is initially at room temperature when the field is turned on, is

17 $$\begin{aligned} T(t) = T_{air} + m_{np} \dfrac{SLP}{\epsilon } \left( 1-e^{-\frac{\epsilon }{C_{susp}}t}\right) . \end{aligned}$$

and, as a straight consequence of Eq. (17), *SLP* may be simply expressed as

18 $$\begin{aligned} SLP = \dfrac{\epsilon }{m_{np}} \dfrac{(T-T_{air})}{\left( 1-e^{-\frac{\epsilon }{C_{susp}}t}\right) }. \end{aligned}$$

In the case of the magnetic field is turned off after the heating, Eq. (16) also provides the temperature response during the cooling process. To this end, assuming $SLP = 0$, the solution for the differential equation, under the condition $T(0) = T_{max}$ as the temperature of the suspension when the field is turned off, is

19 $$\begin{aligned} T(t) = T_{air} + \Delta T_{max} e^{-\frac{\epsilon }{C_{susp}}t}, \end{aligned}$$

in which $\Delta T_{max} = T_{max} - T_{air}$ is the temperature difference between the suspension and the environment when the field is turned off. It is interesting to notice that $T_{max}$ in the cooling process is not necessarily the maximum temperature achieved in the steady state after heating, but it simply corresponds the initial temperature of the suspension anytime when the field is turned off.

Further, we can verify that Eq. (18), by means of Taylor’s expansion $e^{- \frac{\epsilon }{C_{susp}}t} \cong 1 - \frac{\epsilon }{C_{susp}}t$, recovers Eq. (4). This feature reveals Eq. (18) is in fact a generalized form of Eq. (4), both converging in the limit $t\rightarrow 0$, the quasi-adiabatic regime. However, unlike Eq. (4), the validity of Eq. (18) is not restricted to a short time interval after applying the field. At this point, therefore, we may say our approach provides accurate estimates of *SLP* for magnetic nanoparticles under AMF in *non-adiabatic conditions*.

Remarkably, Eq. (17) is a known result, addressed in literature by different groups, that is commonly referred as the phenomenological Box-Lucas equation^19,20,22,23,29^. Nevertheless, although our approach to a system in a non-adiabatic process addressed so far has led us by an well-established path, we emphasize it brought to light the physical meaning of a parameter found in literature that still remained not fully understood so far, the effective thermal conductance expressed in Eq. (15).

### Approaching a system in a non-adiabatic and radiating process

The most striking finding here is that our approach to magnetic hyperthermia for a system in non-adiabatic conditions may be generalized by taking into account also the heat loss rate due to the thermal radiation from the sample to the environment. Hence, we handle with a suspension of magnetic nanoparticles submitted to an alternating magnetic field in *a non-adiabatic and radiating process*.

Generally, it is verified the thermal conductance of the sample holder is much larger than the thermal conductance of the environment, i.e. $\epsilon _{sh} \gg \epsilon _{air,surf}$ and $\epsilon _{sh} \gg \epsilon _{air,top}$. Then, from this assumption which will be proved correct in the next section from the experiment, coming back to Eq. (15), the quantities between parenthesis can be simplified and the effective thermal conductance into the surrounding of the sample may be written as

20 $$\begin{aligned} \epsilon \approx \epsilon _{air,surf} + \epsilon _{air,top} = h_{air} A_{t}, \end{aligned}$$

in which $A_{t} = A_{sh,ext}+A_{top}$. It is worth mentioning that such assumption has been already raised in literature and successfully tested^22,23^, although the reasons justifying its use still remained elusive. Besides, it also leads to $T \approx T_{ext}$, hence the sample holder is assumed to be in thermal equilibrium with the system. With this assumption and its consequences in mind, from the Stefan-Boltzman’s Law^28^, the heat loss rate due to the thermal radiation may be written as

21 $$\begin{aligned} P_{rad} = \sigma \eta A_{t}(T^{4} - T^{4}_{air}), \end{aligned}$$

where $\sigma$ is the Stefan-Boltzmann constant and $\eta$ is the emissivity.

Thereby, taking into account Eqs. (14) and (21) for $P_c$ and $P_{rad}$ respectively, and considering the heat loss rate as

22 $$\begin{aligned} dQ_{susp}/dt = P_{c} + P_{rad}, \end{aligned}$$

from Eq. (6) we may express *SLP* as

23 $$\begin{aligned} SLP = \dfrac{1}{m_{np}} C_{susp} \dfrac{dT}{dt} + \dfrac{1}{m_{np}} \epsilon (T-T_{air}) + \dfrac{1}{m_{np}} \sigma \eta A_{t}(T^{4} - T^{4}_{air}) , \end{aligned}$$

where $\epsilon$ is given by Eq. (20). Such expression corresponds to the differential equation governing the temperature of a suspension of magnetic nanoparticles submitted to an alternating magnetic field, taking into account the contributions of the conduction, convection, and radiation processes to the energy exchange between the system and the environment.

To our knowledge, Eq. (23) does not admit a simple analytical solution. Nevertheless, although it can be solved through numerical procedures, from it we can explore straightly three well-known cases of interest.

The first case consists in the adiabatic approximation. By removing the last two terms of Eq. (23), the heat loss rate of the sample to the environment is completely neglected. As result, the solution of the differential equation recovers Eq. (4), in a similar way to the recovery procedure previously performed from Eq. (18), as expected.

Next, the second case corresponds to the non-adiabatic and non-radiating process. Here, given the heat loss rate due to the radiation is neglected and the last term of Eq. (23) is removed, a linear relation between the heat loss rate and *T* is assumed. Notice the use of Eq. (20) is not fundamental for this second case, so that we can take Eq. (15) to describe $\epsilon$. Then, the solution of the differential equation in the heating process, under $T(0) = T_{air}$ depicting the suspension is initially at room temperature when the field is turned on, is simply Eq. (17).

Finally, the third, in the isothermal case, after long time intervals, $t \rightarrow \infty$, the suspension temperature reaches a maximum value, in which $dT/dt = 0$, and the *SLP* can be expressed simply as

24 $$\begin{aligned} SLP = \dfrac{A_{t}}{m_{np}} \left[ h_{air} (T_{max}-T_{air}) + \sigma \eta (T_{max}^{4} - T^{4}_{air}) \right] , \end{aligned}$$

where $T_{max}$ is the temperature achieved in the steady state. Although this solution is quite useful from the phenomenological perspective, it carries some fundamental problems because the steady state in general is just achieved at high temperatures, in which the hypothesis that *SLP* is independent of temperature is no longer valid^19,22^, and after long time intervals, when issues related with the heating of the own experimental setup often become relevant.

After all, given the stated above, the specific loss power can be directly measured from the experiments. From the theoretical perspective, it is interesting to notice that Eq. (23) can be understood as a general equation, recovering three particular cases of interest, named the adiabatic approximation, the non-adiabatic and non-radiating conditions, and the isothermal case. However, more than that, as aforementioned, as a straight consequence of Eq. (23), *SLP* shall be in principle estimated through numerical solutions. Hence, our approach provides a suitable route to accurate estimates of *SLP* for magnetic nanoparticles under AMF in *a non-adiabatic and radiating process*, as well as to determine $\epsilon$, the effective thermal conductance. In addition, as we demonstrate in the following, it also brings a correction to the estimated experimental results, arisen from the the heat loss rate due to the thermal radiation.

### Comparison with the experiment

To confirm the validity of our theoretical approach, we analysed the thermal behavior of magnetite and magnesium ferrite MNPs dispersed in water submitted to an AMF. Our set of samples here includes superparamagnetic nanoparticles with distinct compositions and different particle sizes (see "Methods" section for details on the magnetic nanoparticles and experiments).

In order to make easier a direct comparison between theory and experiment, as well as to verify the validity of our theoretical approach, we need to make use of conventional units found in literature. To this end, we adopt the temperature in $\mathrm K$, $m_{np}$ in $\mathrm g$, $C_{susp}$ in $\mathrm J/K$, *SLP* in $\mathrm W/g$, $\epsilon$ in $\mathrm W/K$ and *t* in $\mathrm s$.

**Figure 3:**
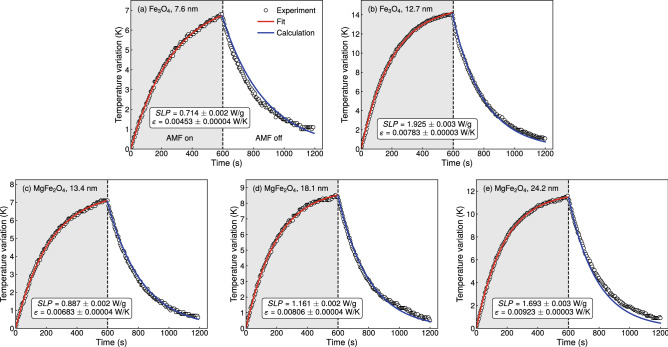
Thermal response of our suspensions. Time evolution of the temperature of our magnetic (**a**,**b**) magnetite and (**c**–**e**) magnesium ferrite nanoparticles dispersed in water. The gray and white zones delimit the time periods corresponding to the heating and cooling processes, in which the suspension is exposed to an alternating magnetic field on and off, respectively. The magnetic hyperthermia experiments were performed with an AMF with frequency of 70.5 kHz and amplitude of 70 Oe. The symbols are the experimental data for the temperature as a function of the time. The red solid line in the heating process is the data fit performed using the solution of Eq. (23) obtained numerically through the Runge–Kutta method. The blue solid line in turn is the numerical calculation for the time evolution of the temperature in the cooling process, obtained solving numerically Eq. (19), in this case assuming *T*(0) as the maximum temperature achieved in the heating process, and the parameter $\epsilon$ obtained previously from the fit. The values of *SLP* and effective thermal conductance into the surrounding of the sample, $\epsilon$, estimated from the fits are reported in Table 1.

Figure 3 depicts the thermal response of our suspensions. Notice the quite-good concordance between experimental data and the fit and numerical calculation obtained from our theoretical approach. Given that $T_{air}$, $m_{np}$, $C_{susp}$, $\sigma = 5.67 \cdot 10^{-8}$ W/(m$^{2}$K$^{4}$), $A_{t} = 0.000910 \pm 0.000003$ m$^{2}$, and $\eta = 0.9$ (this latter a typical value for water and acrylic^28^) are known parameters, we first take into account the experimental data from the heating process; and we fit them using the solution of Eq. (23) obtained numerically through the Runge-Kutta method^30–33^. From this procedure, we estimate here the *SLP* and the effective thermal conductance into the surrounding of the sample, $\epsilon$, which is directly related with the heat transfer coefficient of the air $h_{air}$. Next, considering $SLP = 0$, we calculate the time evolution of the temperature in the cooling process. To this end, here we also employ the Runge-Kutta method, in this case assuming *T*(0) as the maximum temperature achieved in the heating process, and the parameter $\epsilon$ obtained previously from the fit. From this numerical calculation, we corroborate *SLP* and $\epsilon$ obtained from the first fit procedure. Our findings are summarized in Table 1.

From the general point of view, all the main features of the time evolution of the temperature of magnetic nanoparticles dispersed in water submitted to an alternating magnetic field are well described by our approach to the magnetic hyperthermia in a non-adiabatic and radiating process. The tiny differences between experiment and theory, especially when the system is in the cooling process, may be devoted to small changes in the environment and/or modifications in the suspension due to the previous increase of the temperature, which are not taken into account in our model. Further, we do understand the heating of the own experimental setup is yet an important contributor to deviations of the measured temperature with respect to the expected thermal behavior for after long time intervals. Indeed, we performed experimental measurements considering pure water as the probed system, and our findings reveals us an increase in the water sample temperature of around 1 K after 1200 s, a temperature variation assumed as acceptable from the experimental perspective^19,20,23^.

We obtain here consistent *SLP* results for our nanoparticles and raise fundamental issues regarding the estimated effective thermal conductance into the surrounding of the sample.

An interesting feature here is the *SLP* value itself, as well as in its accuracy, i.e. the standard deviation of the values estimated with our approach to the magnetic hyperthermia in a non-adiabatic and radiating process. Specifically, we find values between 0.714 and 1.925 W/g for our suspensions, and we verify a clear raise of the specific loss power with the particle size, as expected. In addition, from Table 1, we can check the standard deviations of *SLP* fall into the range between 0.002 and 0.003 W/g.

**Table 1:** Summary of our findings.

Composition	Particle size (nm)	Adiabatic approach	Non-adiabatic approach		Non-adiabatic and radiating process		
		SLP (W/g)	SLP (W/g)	${\epsilon }$ (W/K)	${{SLP}}$ (W/g)	${\epsilon }$ (W/K)	${{h}}_{air}$ (W/(m$^{2}$K))
$\mathbf{Fe} _3\mathbf{O} _\mathbf{4}$	$7.6\pm 0.2$	$0.67\pm 0.01$	$0.717\pm 0.002$	$0.00939 \pm 0.00004$	$0.714\pm 0.002$	$0.00453 \pm 0.00004$	$4.98 \pm 0.06$
$\mathbf{Fe} _3\mathbf{O} _\mathbf{4}$	$12.7\pm 0.2$	$1.60\pm 0.02$	$1.940\pm 0.003$	$0.01293 \pm 0.00003$	$1.925\pm 0.003$	$0.00783 \pm 0.00003$	$8.61 \pm 0.06$
$\mathbf{MgFe} _2\mathbf{O} _\mathbf{4}$	$13.4\pm 0.3$	$0.86\pm 0.01$	$0.891\pm 0.002$	$0.01171 \pm 0.00004$	$0.887\pm 0.002$	$0.00683 \pm 0.00004$	$7.51 \pm 0.06$
$\mathbf{MgFe}_2\mathbf{O}_\mathbf{4}$	$18.1\pm 0.2$	$1.07\pm 0.01$	$1.167\pm 0.002$	$0.01299 \pm 0.00004$	$1.161\pm 0.002$	$0.00806 \pm 0.00004$	$8.87\pm 0.07$
$\mathbf{MgFe}_2\mathbf{O}_\mathbf{4}$	$24.2\pm 0.2$	$1.43\pm 0.01$	$1.704\pm 0.003$	$0.01425\pm 0.00003$	$1.693\pm 0.003$	$0.00923\pm 0.00003$	$10.15\pm 0.07$

The average particle size is estimated by TEM. The experimental thermal quantities for our magnetite and magnesium ferrite nanoparticles are estimated by fitting, using the theoretical approaches, from magnetic hyperthermia experiments performed with alternating magnetic field with frequency of 70.5 kHz and amplitude of 70 Oe. Specifically, we estimate *SLP* and $\epsilon$, and consequently $h_{air}$ from Eq. (20), from our approach describing magnetic nanoparticles under AMF in a non-adiabatic and radiating process, as well as, for comparison, the *SLP* from the adiabatic model, and *SLP* and $\epsilon$ from the approach considering non-adiabatic conditions.

The dependence of the *SLP* with intrinsic parameters of the sample, such as composition, average diameter, size distribution, morphology, and crystalline structure of the particles^13,24,34^, as well as viscosity of the fluid carrier^15,35^, has been previously verified by numerous groups. Further, it is well-known the *SLP* is dependent on the AMF, evolving in a different form with frequency and amplitude. In this case, for instance Hergt and colleagues^36^ have shown for aqueous suspensions of magnetite, with nanoparticles having sizes of 6 and 8 nm, *SLP* values 0.1 and 21 W/g, respectively, for a field with frequency of 300 kHz and amplitude of 82 Oe; Zhang and coworkers^15^ in turn have estimated for a similar system, with coated nanoparticles of 16 nm and uncoated ones of 50 nm, *SLP* values 55 and 4.5 W/g, respectively, for a field with 55 kHz and 200 Oe; at last, Barati and workmates have disclosed for aqueous suspension of MgFe$_2$O$_4$ nanoparticles, with sizes from 8.4 up to 21 nm, *SLP* values falling within the range between 8.4 and 12 W/g for a field with 279 kHz and 100 Oe^37^. Therefore, our results are also compatible with distinct findings reported literature.

To highlight our achievements with the approach to magnetic hyperthermia in a non-adiabatic and radiating process, we also carried out an analysis of our experimental results considering the quasi-adiabatic method, thus employing Eq. (4), as well as the approach for magnetic nanoparticles under AMF in non-adiabatic conditions, then using Eq. (17) for the fits. The results obtained from this analysis procedure are also presented in Table 1 to make easier the comparison with our findings.

In this respect, we first perform the fits with the quasi-adiabatic method considering the temperature variation of the suspension during the first 30 s of the experiment. Besides obtaining underestimated *SLP* results, between $\sim 0.67$ and $\sim 1.60$ W/g, the accuracy with the quasi-adiabatic method through Eq. (4) is substantially worse, with standard deviation values being one order of magnitude higher than those found from both non-adiabatic model and our approach to a non-adiabatic and radiating process. Curiously enough, at first glance, the *SLP* results estimated with our approach might mislead us, suggesting they are roughly similar to the ones found for the non-adiabatic approach in Table 1. However, despite they behave in a remarkably similar manner, a closer examination shows us the *SLP* values are slightly smaller than those obtained with Eq. (17). We interpret this tiny difference as the first fingerprint of the heat loss rate due to the thermal radiation influencing the balance of the fit.

The most striking finding resides in the $\epsilon$, the parameter defined by Eq. (15) and named here as the effective thermal conductance into the surrounding of the sample. The $\epsilon$ values obtained from the fits of the experimental data in the heating process using Eqs. (17) and (23) are shown in Table 1 too. Remarkably, the $\epsilon$ results estimated with our approach are also systematically smaller than those obtained from the non-adiabatic approach, and all of them present similar accuracy. In this sense, we demonstrate that our approach to magnetic hyperthermia in a non-adiabatic and radiating process brings a correction to the *SLP* and $\varepsilon$ values obtained from experiment, arisen from the heat loss rate due to the thermal radiation.

Although some type of correlation between the *SLP* and $\epsilon$ may be raised from the values depicted in Table 1, this issue is not fully understood and still remains under investigation. We understand the tiny variations in the values of the effective thermal conductance may be devoted to the fluctuations promoted by changes in the environment (which by the way may be related with the convection process too), modifications in the suspension due to the previous increase of the temperature, difference in surface area between the samples, as well as limitations of the own experimental setup.

In particular, we find the average $\epsilon$ parameter is 0.00730 W/g. It is worth mentioning that we have also performed tests considering distinct samples and fields having different amplitudes and frequencies, not addressed here; and all experiments uncover roughly similar $\epsilon$ parameter, although we may identify some dependence of its value with the temperature, a fact possibly attributed to the convective effects. Thereby, the small relative inaccuracy suggests this parameter is independent of the sample and/or magnetic field; but it is intrinsically related to the environment into the surrounding of the sample and the surface area of contact between the sample and environment, as expected whether Eq. (15) is indeed valid.

From Eqs. (11), (12) and (13), and considering the dimensions of our sample holder, the thermal conductivity of acrylic $\kappa _{sh} = 0.2$ W/(m$\cdot$K)^38^, and the heat transfer coefficient of the air $h_{air} = 8$ W/(m$^{2}\cdot$K)^28^, we obtain the respective thermal conductances $\epsilon _{sh} = 0.0910$ W/K, $\epsilon _{air,surf} = 0.0048$ W/K and $\epsilon _{air,top} = 0.0024$ W/K. In regard to the effective thermal conductance into the surrounding of the sample, from Eq. (15) we then verify $\epsilon = 0.0071$ W/g, which is in very good agreement with the findings achieved with our approach to magnetic hyperthermia in a non-adiabatic and radiating process. Hence, we bring experimental evidence that supports the validity of such description for the effective thermal conductance.

From the experimental values aforementioned for the thermal conductances, we also provide clear evidence that the thermal conductance of the sample holder is much larger than the thermal conductance of the environment, $\epsilon _{sh} \gg \epsilon _{air,surf}$ and $\epsilon _{sh} \gg \epsilon _{air,top}$. It is important to point out that, in the construction of our approach to magnetic hyperthermia in a non-adiabatic and radiating process, we considered such assumption and simplified Eq. (15), then obtaining Eq. (20). We find $\epsilon = 0.0073$ W/K from this latter, a value very close to the one measured from Eq. (15). This concordance allows us to consider our assumption and simplification are plausible.

Remarkably, Eq. (20) describing the effective thermal conductance into the surrounding of the sample is exclusively dependent on the thermal conductance of the air. From this perspective, we may infer such conductance associated to the convection process around the sample/sample holder has the main role in the dynamics, when compared to that related to conduction and radiating processes.

Last but not least, we estimate the heat transfer coefficient of the air using Eq. (20) either. These results are also shown in Table 1. Noticeably, the $h_{air}$ values fall into the range known for gases in free convection reported in literature^28^. This concordance between our findings and well established values for the heat transfer coefficient can be understood as an additional test of consistency to our approach to magnetic hyperthermia in a non-adiabatic and radiating process.

After all, the quantitative agreement of predictions with experimental results does confirm the robustness of our theoretical approach. Hence, we provide physical meaning to a parameter found in literature that still remained not fully understood, named here the effective thermal conductance into the surrounding of the sample, as well as bring to light how they can be obtained experimentally. In addition, our findings place the theoretical approach to magnetic hyperthermia based on thermodynamics concepts, that takes into account the interaction of the system with the environment, as a sharp tool for the determination of an accurate, reliable specific loss power value for magnetic nanoparticles under AMF, as well as for estimating $\epsilon$, the effective thermal conductance, from a non-adiabatic and radiating process.

## Discussion

In summary, we have performed a theoretical and experimental investigation of the magnetic hyperthermia in suspensions of magnetic nanoparticles. Here we have proposed a theoretical approach to magnetic hyperthermia from a thermodynamic point of view. To test the robustness of the approach, we have performed hyperthermia experiments and analyse the thermal behavior of magnetite and magnesium ferrite magnetic nanoparticles dispersed in water submitted to an alternating magnetic field.

By comparing experiment and theory, the model has allowed us to obtain the specific loss power of a suspension submitted to an alternating magnetic field from a non-adiabatic and radiating process. Remarkably, we have verified our approach enhances the accuracy in the determination of this quantity, when compared to the quasi-adiabatic method. In addition, if compared to the results obtained through the non-adiabatic model, our approach brings a correction to the *SLP* and $\epsilon$ values obtained from experiment, arisen from the the heat loss rate due to the thermal radiation.

We have also provided physical meaning to a parameter found in literature that still remained not fully understood so far. Specifically, we have addressed the effective thermal conductance, bringing to the light how it can be obtained from experiment. In this respect, we have yet provided evidences that effective thermal conductance is intrinsically related to the environment into the surrounding of the sample and the surface area of contact between the sample and environment. Morever, we have verified that $\epsilon$ results obtained from our approach are systematically smaller than those obtained from the non-adiabatic model, suggesting those latter are overestimated.

After all, it is worth remarking the quantitative agreement of predictions with experimental results has confirmed the validity of our theoretical approach. Thereby, our findings place the theoretical approach to magnetic hyperthermia based on thermodynamics concepts that takes into account the interaction of the system with the environment as a sharp tool for the determination of an accurate, reliable specific loss power value for magnetic nanoparticles under AMF, as well as for estimating $\epsilon$, the effective thermal conductance, from a non-adiabatic and radiating process. More than that, we show our approach also brings a correction to the estimated experimental results, thus demonstrating the importance of the heat loss rate due to the thermal radiation in magnetic hyperthermia.

## Methods

### Set of samples

For the study, we prepared a set of 5 samples. Two of them are magnetite $\mathrm{Fe}_{3}\mathrm O_{4}$ nanoparticles, with average particle size of 7.6 and 12.7 nm, synthesized by co-precipitation considering distinct proportions of precursor reagents^39,40^. The other three samples are magnesium ferrite $\mathrm{MgFe}_{2}\mathrm O_{4}$ nanoparticles, produced by sol-gel followed by calcination at the selected temperatures of 400, 500 and $600^{\circ }$C for 2 h^41,42^. These latter have average particle size of 13.4, 18.1, and 24.2 nm, respectively. Thereby, our set is composed by nanoparticles having distinct compositions and different particle sizes.

### Structural and morphological characterization

The structural and morphological properties of the nanoparticles were verified by X-ray diffractometry (XRD) and transmission electron microscopy (TEM). The diffraction measurements were performed with a Rigaku MineFlex II diffractometer, and the results were refined by Rietveld method using the software MAUD, thus allowing the identification of the phase, and providing lattice parameters and crystallite size. TEM images were acquired with a JEM-1011 transmission electron microscope and analysed using the software ImageJ, then informing the phase, particle shape and distribution of the average particle diameter.

**Figure 4:**
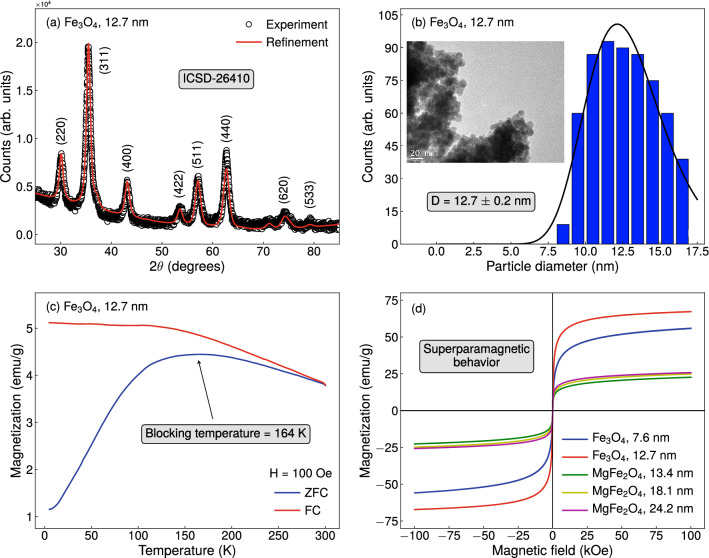
Structural, morphological and magnetic properties of our magnetite and magnesium ferrite nanoparticles. (**a**) X-ray diffraction pattern with Rietveld refinement, (**b**) transmission electron microscopy image with histogram of particle size distribution fitted with a log-normal function, and (**c**) ZFC and FC magnetization curves acquired with probe magnetic field of 100 Oe for the magnetite sample with average particle diameter of 12.7 nm, as representative examples of our findings for the investigated nanoparticles. (**d**) Isothermal magnetization curves measured at room temperature for the magnetite and magnesium ferrite samples with distinct particle sizes.

Figures 4a,b bring representative examples of the results obtained from the structural and morphological characterization. From the XRD experiments, we first confirm our samples are single phase. Specifically, diffraction peaks for the magnetite samples are well indexed with the standard pattern ICSD-26410, and can be associated to the (220), (311), (400), (422), (511), (440), (620), (533) planes. These findings are in very good agreement with reports found in the literature^43–45^. The results for the magnesium ferrite in turn, not shown here, are in quite-good concordance with ICSD-152468 and with findings previously reported by different groups^46–49^, presenting peaks located at $2\theta$ ranging from $28^\circ$ to $80^\circ$, which are associated with the (220), (311), (222), (400), (422), (511), (440), (620) and (533) planes of the MgFe$_{2}$O$_{4}$. For both compositions, the patterns raise fingerprints of phases having cubic symmetry and Fd:3m space group. Rietveld refinement yet informs us the crystallite size, confirming our procedures as promising routes to the production of pure nanoparticles with specific sizes. All these findings are corroborated by TEM. TEM images also show the particles are quite uniform, having approximately spherical geometry, despite the formation of the clusters. The histograms of particle size distribution fitted with a log-normal function confirm the aforementioned average particle diameter values between 7 and 25 nm. Table 1 discloses specifically our findings on the particle size for each sample.

### Magnetic characterization

The magnetic characterization of the nanoparticles was performed using a Quantum Design Dynacool Physical Property Measurement System through zero-field-cooled (ZFC) and field-cooled (FC) magnetization measurements, acquired in the range of temperature between 4 and 300 K with probe magnetic field of 100 Oe, as well as via isothermal magnetization curves acquired at selected temperatures.

Figure 4c shows a representative example of the ZFC and FC magnetization curves measured for our samples. All the main features of both curves representing the dependence of the magnetization with temperature are well understood. From our concern at this moment, we highlight the ZFC curves are characterized by a broad cusp, whose location of the maximum defined the system blocking temperature, in which the nanoparticles exhibit a magnetic transition between the superparamagnetic and blocked states. For our set, we find blocking temperature values within the range between 135 and 190 K. Similar results are found in literature for both, magnetite^43,44^ and magnesium ferrite^49–53^ nanoparticles. In this sense, our samples are superparamagnetic at room temperature. Figure 4d presents the magnetization curves acquired for our magnetite and magnesium ferrite samples with distinct particle sizes. Remarkably, all samples exhibit a typical behavior of a soft magnetic material. Below the blocking temperature, the isothermal magnetization curves, not shown here, exhibit hysteresis, as expected. At room temperature, we observe s-shaped curves, with low remanent magnetization and small values of coercive field, being well described by a Langevin function, characterizing the superparamagnetic state.

### Magnetic hyperthermia experiments

The calorimetric measurements were carried out with a homemade experimental setup. The system consists basically of two parts, one responsible by generating of the AMF and another by the detection of the sample temperature. The first one is composed of a parallel LC resonant circuit^54^, which includes the solenoid and provides a homogeneous sinusoidal magnetic field with frequency of 70.5 kHz and amplitude of 70 Oe. We took special care to minimize effects due to Joule losses during the measurements. In this respect, a cooling system is responsible by keeping the solenoid at room temperature. The second part of the system consists in an Extech HD300 infrared thermometer, which allows us to perform precise acquisitions of the sample temperature. All the measurements were performed in suspension samples, consisting of 100 mg of nanoparticles dispersed in 0.6 mL of distilled water, inside a cylindrical sample holder made of acrylic. Specifically, we divided the experiment in two stages. In the first stage, once the suspension was at room temperature, we turned on the AMF and started acquiring the sample temperature. After recording the temperature in the heating process during 600 s, the second stage begins when the field is turned off, and we kept the temperature measure for an additional period of 600 s during the cooling process.
